# HSP90 Inhibition Enhances Antimitotic Drug-Induced Mitotic Arrest and Cell Death in Preclinical Models of Non-Small Cell Lung Cancer

**DOI:** 10.1371/journal.pone.0115228

**Published:** 2014-12-26

**Authors:** Brenda C. O'Connell, Katie O'Callaghan, Bonnie Tillotson, Mark Douglas, Nafeeza Hafeez, Kip A. West, Howard Stern, Janid A. Ali, Paul Changelian, Christian C. Fritz, Vito J. Palombella, Karen McGovern, Jeffery L. Kutok

**Affiliations:** Infinity Pharmaceuticals, Inc., Cambridge, MA, United States of America; University of Illinois at Chicago, United States of America

## Abstract

HSP90 inhibitors are currently undergoing clinical evaluation in combination with antimitotic drugs in non-small cell lung cancer (NSCLC), but little is known about the cellular effects of this novel drug combination. Therefore, we investigated the molecular mechanism of action of IPI-504 (retaspimycin HCl), a potent and selective inhibitor of HSP90, in combination with the microtubule targeting agent (MTA) docetaxel, in preclinical models of NSCLC. We identified a subset of NSCLC cell lines in which these drugs act in synergy to enhance cell death. Xenograft models of NSCLC demonstrated tumor growth inhibition, and in some cases, regression in response to combination treatment. Treatment with IPI-504 enhanced the antimitotic effects of docetaxel leading to the hypothesis that the mitotic checkpoint is required for the response to drug combination. Supporting this hypothesis, overriding the checkpoint with an Aurora kinase inhibitor diminished the cell death synergy of IPI-504 and docetaxel. To investigate the molecular basis of synergy, an unbiased stable isotope labeling by amino acids in cell culture (SILAC) proteomic approach was employed. Several mitotic regulators, including components of the ubiquitin ligase, anaphase promoting complex (APC/C), were specifically down-regulated in response to combination treatment. Loss of APC/C by RNAi sensitized cells to docetaxel and enhanced its antimitotic effects. Treatment with a PLK1 inhibitor (BI2536) also sensitized cells to IPI-504, indicating that combination effects may be broadly applicable to other classes of mitotic inhibitors. Our data provide a preclinical rationale for testing the combination of IPI-504 and docetaxel in NSCLC.

## Introduction

The mitotic, or spindle assembly checkpoint helps maintain genomic integrity by preventing the missegregation of chromosomes. A highly orchestrated surveillance system composed of numerous proteins detects unattached kinetochores, or lack of proper tension across the mitotic spindle, triggering the so-called “checkpoint response”, which leads to mitotic arrest. Normal cell division requires successful passage through the mitotic checkpoint. Failure to satisfy checkpoint requirements within a relatively short timeframe (1–2 days) can result in aneuploidy, mitotic catastrophe, or mitotic slippage followed by a variety of cell fates including cell death, senescence, or endoreduplication [1]. While the mechanisms by which prolonged mitosis leads to cell death are unclear, a role for the anti-apoptotic BCL2 family members has been reported [2]. During prolonged mitotic arrest, cyclin-cyclin dependent kinase (CDK) proteins phosphorylate *BCL2* family members including BCL2, BCL-XL, and MCL1. Phosphorylation of BCL2 and BCL-XL results in the release of pro-apoptotic proteins BAX/BAK; whereas phosphorylation of MCL1 creates a recognition site for the E3 ligase, APC/CDC20, targeting it for proteasomal degradation. Functional redundancy is likely to exist among the *BCL2* family members in mediating the cell death response to prolonged mitosis.

Antimitotic drugs that target microtubule dynamics (MTAs) are widely used in the clinic to treat a broad range of cancers. These include microtubule stabilizing agents, (taxanes, including docetaxel and paclitaxel, and epothilones) and microtubule destabilizing agents (including vinca alkaloids such as vincristine and vinblastine) [3]. In addition, Maytansines (DM1, DM4) and Auristatins (MMAE, MMAF) interact with the vinca binding site on tubulin and are commonly used as the toxin attached to antibody drug conjugates [4]. While dividing tumor cells are susceptible to MTAs, other microtubule-dependent cellular processes such as vesicle trafficking, neuronal transport, and cytoskeletal integrity are also disrupted, leading to undesired side effects including neurotoxicity and myeloid toxicity [5]. In an effort to overcome these side effects, antimitotic drugs that target the spindle motor proteins (KSP, Eg5) or mitotic kinases (PLK1, Aurora Kinase A, Aurora Kinase B) are being developed, but have met with limited success so far in the clinic [6]. HSP90 is a molecular chaperone that is responsible for the proper folding of numerous client proteins, including many oncogenes and mutated tumor suppressors [7]. The HSP90 inhibitor IPI-504 has demonstrated antineoplastic activity in several preclinical models of cancer, providing rationale for its further clinical development [7], [8], [9], [10], [11]. Interestingly, synergistic activity between HSP90 inhibition and taxanes has been observed in preclinical models of NSCLC [12] and HSP90 inhibitors have been evaluated in combination with docetaxel in clinical studies of NSCLC (NCT01646125, NCT01348126, NCT01798485, NCT01362400). We identified a subset of NSCLC cell lines in which IPI-504 and docetaxel act in synergy to enhance cell death in vitro and inhibit tumor growth in vivo. Because the precise molecular basis for this synergy has not been determined, we investigated the molecular mechanism of action (MOA) of IPI-504 in combination with docetaxel and other antimitotics. Our studies revealed an MOA involving a checkpoint dependent lengthening of mitosis. Further, we identified APC/C components as potential novel HSP90 client proteins, partially responsible for the drug synergy.

## Materials and Methods

### Ethics statement

This study was conducted in accordance with the recommendations in the Guide for the Care and Use of Laboratory Animals printed by the National Research Council of the National Academics. The protocol was approved by the Institutional Animal Care and Use Committee (IACUC) at Infinity Pharmaceuticals, Inc. Animals were euthanized by CO_2_ inhalation according to IACUC guidelines. Every effort was made to minimize animal suffering.

### Cell lines

Human NSCLC cell lines H292, A549, H522, H1993, H1793 obtained from American Type Culture Collection were maintained for several passages under 5% CO_2_ at 37°C in RPMI 1640 media supplemented with 10% fetal bovine serum (Sigma-Aldrich).

### Animal studies

Five- to six-week old male NCR nu/nu athymic mice were purchased from Taconic Farms. Xenografts were generated by subcutaneous implantation of 1 to 5×10^6^ cells into the right flank of mice, and treatment was initiated when tumors reached an average volume of 120 to 300 mm^3^. IPI-504 was administered intra-peritoneally twice per week at a dose of 50 mg/kg. Docetaxel (McKeeson Pharmaceuticals) was dosed once per week at 15 mg/kg (H1993, A549 and H292 xenograft models) or 5 mg/kg (H292 xenograft model) by intraperitoneal injection.

Tumors were measured three times per week using digital calipers and tumor volume was calculated using the formula: (length x width^2^)/2. Results are presented as average tumor volume ± standard error of the mean (SEM).

### Cell proliferation

Cells were seeded in 96-well plates at a density of 5000 cells/well 24 h prior to treatment with combinations of IPI-504 and docetaxel as indicated. Cell proliferation was measured by Alamar Blue (Life Technologies) or Cell Titer Glo (Promega). Cell death was measured by the percentage of 7AAD positive cells (Guava Viacount Flex) or by the percentage of cleaved caspase 3 positive cells in a luminescence based assay (Promega; Caspase-Glo3/7).

### Synergy studies

Combination indexes (CI) were determined by the method of Chou and Talalay [13] using fixed and non-fixed drug ratios and CalcuSyn software (Biosoft). CI values <1 indicate synergy, with values <0.5 indicating robust synergy.

### Immunoblotting/Immunoprecipitation

For immunoblotting, cells were lysed in RIPA lysis buffer (Sigma-Aldrich) supplemented with protease inhibitors (Roche) and phosphatase inhibitors (HALT; Thermo Fisher Scientific). For HSP90 immunoprecipitations, cell pellets were harvested post drug treatment in non-detergent lysis buffer (50 mM Tris pH 7.4, 20 mM NaCl, 2 mM MgCl_2_, 1 mM EDTA, 10% glycerol, protease inhibitor tablet (Roche) and phosphatase inhibitors (Thermo Fisher Scientific)) followed by three sequential freeze thaw cycles for lysis. Immunoprecipitations were performed overnight at 4°C using an HSP90 monoclonal antibody (Santa Cruz) and Sepharose GammaBind G beads (GE Healthcare).

### Stable Isotope Labeling of Amino Acids in Culture (SILAC) labeling and mass spectrometry

Metabolic labeling of H292 cells was carried out with normal arginine and lysine or heavier isotopic variants of the two amino acids L-Lysine-HCl [^13^C_6_], L-arginine-HCl [^13^C_6_, ^15^N_4_] using Invitrogen's SILAC-Flex Media kit and heavy arginine purchased from Thermo Fisher Scientific. To decrease sample complexity, HSP90 immunoprecipitations were performed on drug treated cells and proteins were separated by 1D-SDS-PAGE. Proteins from gel slices were digested using porcine trypsin and analyzed by LC-MS/MS. Peptides were cleaned up and concentrated using C18 stage tips (Proxeon) and separated using online C18 reverse-phase nanoscale liquid chromatography tandem mass spectrometry on a Surveyor MS pump connected to a LTQ-Orbitrap XL (Thermo Fischer Scientific) using a 2 h linear gradient. Fragmentation of the top 10 peptides in each sample was performed by collision-induced dissociation. Raw MS files from LTQ-orbitrap were analyzed using MaxQuant (version 1.2.2.4) [14]. MS/MS spectra were searched against the decoy IPI-human database version 3.68 using the Andromeda search engine. A false discovery rate of 0.01 was used on both the peptide and protein levels.

### RNAi studies

H292 cells were transfected with 30 nM siRNA using RNAimax (Invitrogen). siRNAs were purchased from Thermo Fisher Scientific as ON-TARGET plus SMART pools (mix of 4 individual siRNAs per gene). SMART pool siRNA sequences are: non-targeting (scrambled) control: UGGUUUACAUGUCGACUAA, UGGUUUACAUGUUGUGUGA, UGGUUUACAUGUUUUCUGA, UGGUUUACAUGUUUUCCUA; ANAPC3:GGAAAUAGCCGAGAGGUAA, CAAAAGAGCCUUAGUUUAA, AAUGAUAGCCUGGAAAUUA, GCAUAUAGACUCUUGAAAG; and ANAPC4: GCCAGAAAGUUUACUCAUA, GAUGAACAGUGUAGUGCUA, ACACGUAGAUUGUUCAAAU, CGCUUUAGCUCCAGAGAUA. Four h post transfection, cells were seeded into 96-well plates, treated with a dose titration of docetaxel and harvested for flow cytometry (pH3) or cell proliferation (7AAD) 30 h or 72 h post drug treatment, respectively.

### Flow cytometry

Cell pellets were collected by trypsinization, fixed in 4% paraformaldehyde at 37°C for 15 min, placed on ice for 5 min, and then pelleted and resuspended in ice cold methanol for 30 min on ice. Next, cell pellets were washed in 1% bovine albumin serum (BSA) in PBS twice followed by incubation with a FITC-conjugated pH3 antibody for 2 h RT. Cell pellets were washed twice with 1% BSA/PBS and resuspended in 2 µg/mL Hoechst (Molecular Probes)/PBS at 37°C for 15 min. Stained cells were analyzed using a BD LSRII Fortessa flow cytometer. FITC positive cells were measured at a wavelength of 488 nm, and DNA content was measured by Hoechst staining using a UV laser. Data analysis was performed using FlowJo software (V7.6.3).

### Microscopy

Phase contrast images were captured using a Nikon Eclipse TE2000-S microscope and Spot software for image acquisition (V4.7).

### Mitotic shake-off

Mitotic cells were harvested by manual tapping of the flask to dislodge mitotic cells into suspension. Mitotic cells were isolated from the media by centrifugation (1500 rpm, 5 min), lysed in RIPA buffer and incubated in the presence or absence of alkaline phosphatase (Sigma-Aldrich).

### Antibodies and reagents

Antibodies for HSP90 (Santa Cruz), HSP70 (Santa Cruz), Cyclin B (BD Biosciences), Securin (Abcam), ANAPC3 (Bethyl Laboratories), ANAPC4 (Bethyl Laboratories), Aurora kinase B (Bethyl Laboratories), MCL1 (Cell Signaling Technology), GAPDH (Cell Signaling Technology), Glucocorticoid Receptor (Cell Signaling Technology), and FITC-S10 phospho-Histone H3 (Cell Signaling Technology) were used for immunoprecipitation, immunoblotting and flow cytometry. Aurora A/B inhibitor (ZM447439) was purchased from EMD Millipore and PLK1 inhibitor (BI2536) was purchased from SelleckChem. IPI-504 was synthesized at Infinity Pharmaceuticals, Inc.

## Results

### IPI-504 and docetaxel in combination suppress tumor growth in NSCLC tumor xenograft models

**Figure 1:**
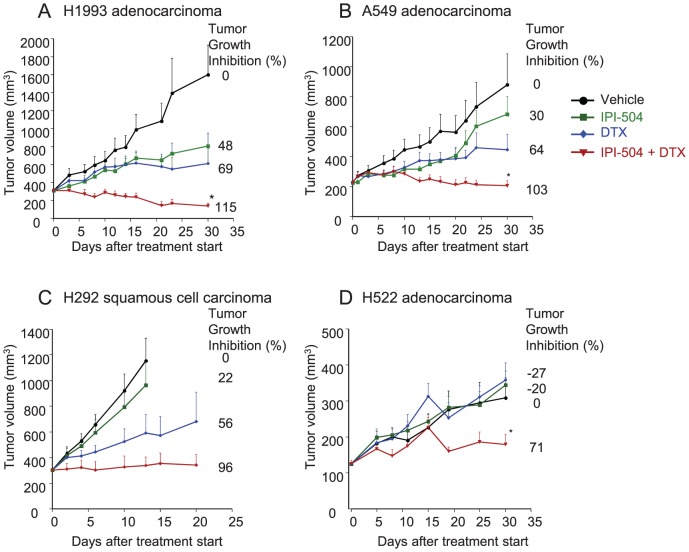
Combination of IPI-504 and docetaxel (DTX) improves efficacy in NSCLC xenograft models. NCR nu/nu homozygous male mice were subcutaneously implanted with (A) H1993, (B) A549, (C) H292 and (D) H522 cells and treated with DMSO vehicle (black circles), IPI-504 (green squares), DTX (blue diamonds), or the combination of IPI-504 and DTX (red triangles). IPI-504 was administered by IP injection at a dose of 50 mg/kg, twice a week for a total of 6 doses. DTX was administered at 5 mg/kg (H522) or 15 mg/kg (H1993, A549, H292) by IP injection, weekly for a total of 3 doses. Numbers on graphs represent average percent tumor growth inhibition relative to vehicle-treated arm. Where indicated, * denotes statistical significance (p<0.05) between combination treatment and DTX treatment arms measured at day 30 (H1993, H549, H522) using Student's T-test.

To identify NSCLC cell lines demonstrating in vivo sensitivity to the combination of IPI-504 and docetaxel, mice bearing xenograft tumors (H1993, A549, H522 and H292) were treated with vehicle, 50 mg/kg IPI-504 alone, 5 or 15 mg/kg docetaxel alone, or a combination of 50 mg/kg IPI-504 and 5 or 15 mg/kg docetaxel. Treatment with IPI-504 alone inhibited tumor growth relative to vehicle in H1993, A549, and H292 xenografts (22–48%) but had no activity in H522 xenografts (Fig. 1). Single-agent activity was observed upon treatment with docetaxel, resulting in growth inhibition compared to vehicle in H1993, A549 and H292 xenografts (56–69%) (Fig. 1A–C). In contrast to the single-agent activity, tumor regression was observed in response to combined IPI-504 and docetaxel in H1993 and A549 xenograft models (Fig.s. 1A and B). Tumor growth inhibition was enhanced by the combination compared to either single agent alone in H292 xenograft tumors (Fig. 1C). Because of the strong single-agent activity in the H522 xenograft model, docetaxel was dose reduced from 15 to 5 mg/kg for the combination study. The combination of 5 mg/kg docetaxel and 50 mg/kg IPI-504 resulted in 71% tumor growth inhibition as compared to vehicle (Fig. 1D). These data indicate potential synergistic effects of IPI-504 and docetaxel on growth inhibition in preclinical models of NSCLC.

### IPI-504 and docetaxel in combination show in vitro synergy in NSCLC cell lines

**Figure 2:**
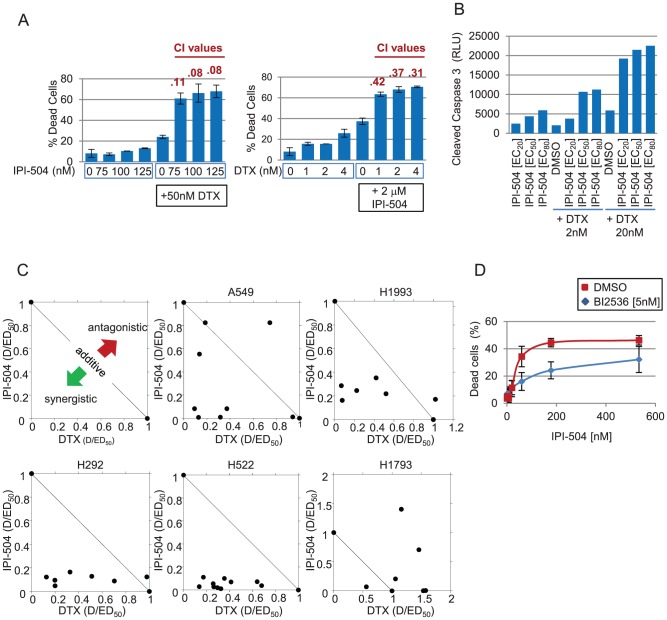
In vitro synergy observed in NSCLC cell lines. (A) Cell death was measured 48 h post treatment with non-fixed drug ratio combinations of IPI-504 and docetaxel (DTX) by 7AAD in H292 cells. Combination indices (CI) values were calculated using CalcuSyn software (Biosoft) with values <0.5 indicative of strong synergy. Error bars represent standard deviation (n = 4). Values for all combination treatments were statistically significant compared to single agent treatments as determined by Student's T-test (p<.01). (B) Cell death was measured 30 h post drug treatment in H1993 cells using the Caspase-Glo3/7 luminescence assay. Representative data are shown (n = 2). (C) Cells were treated with fixed drug ratios of IPI-504 and DTX for 72 h; cell proliferation was measured with Alamar Blue. Shown are normalized isobolograms. D =  dose, ED_50_ =  dose required to attain 50% growth inhibition. Points on the graph refer to the ratio of D/ED_50_ for DTX on the x-axis vs D/ED_50_ for IPI-504 on the y-axis. Data points which fall on the diagonal represent additivity; above the diagonal, antagonism; below the diagonal, synergy. (D) Treatment with PLK1 inhibitor (BI2436) sensitizes H292 cells to IPI-504. H292 cells were treated for 72 h with a dose titration of IPI-504 alone (blue diamonds) or in combination with 5 nM BI2536 (red squares) followed by cell death assay (7AAD). Error bars represent standard deviation (n = 2).

To investigate the MOA of enhanced in vivo tumor inhibition with combined IPI-504 and docetaxel, the effect of the combination on cell death and cell proliferation was determined in vitro. For cell death studies in H292 cells, combination indices were calculated using the non-fixed drug ratio method of Chou and Talalay, with CI values <1.0 indicative of synergy [13]. Doses of IPI-504 (75 to 125 nM) effective for growth inhibition (S1A Fig.) were largely ineffective in yielding a cytotoxic response above control in H292 cells (Fig. 2A, left panel). However, combining IPI-504 (75 to 125 nM) with 50 nM docetaxel increased the H292 cell death from 24% (docetaxel alone) to 61–68% (combination) with CI values indicative of strong synergy (CI<0.2). Similarly, combining docetaxel (1 to 4 nM) with 2 µM IPI-504 increased the H292 cell death from 37% (IPI-504 alone) to 63 to 71% (combination) with CI values indicative of synergy (CI<0.5) (Fig. 2A, right panel).

In H1993 cells, combinations of low (2 nM) or high (20 nM) doses of docetaxel with doses of IPI-504 representing the EC_20_ (14 nM), EC_50_ (59 nM), or EC_80_ (255 nM) for growth inhibition (S1A Fig.) were examined for cell death using a Caspase-Glo3/7 assay. Increases of 2- to 4-fold in caspase activity were observed with the drug combination relative to single agent responses for all but the lowest-dose combination of 14 nM IPI-504 and 2 nM docetaxel (Fig. 2B).

For cell proliferation studies, a panel of cell lines was treated with fixed drug ratios. A synergistic response to drug combination was observed in all four cell lines for which combination effects were previously observed in vivo (Fig. 2C; A549, H1993, H292, and H522). There was no indication of a synergistic effect of the drug combination in H1793 cells, suggesting that certain cell lines may not be responsive to this specific combination (Fig. 2C). Unfortunately, confirmatory xenograft studies were not possible with H1793, as the cells did not grow in either immunocompromised NCR nu/nu or NOD/SCID mice. H292 cells were chosen for the majority of the subsequent mechanistic studies, since they displayed the strongest and most consistent synergistic response to drug combination in vitro, however, other cell lines were also studied in specific cases. Overall, these findings indicate that some NSCLC cell lines show marked decreases in cell proliferation and increases in apoptotic cell death with combined IPI-504 and docetaxel treatment compared to the single agents alone.

Inhibitors designed to target mitotic kinases, such as PLK1, represent an alternative class of antimitotics to MTAs. PLK1 is an important regulator of mitosis and a known HSP90 interacting protein [15]. A PLK inhibitor (BI2536) exhibiting 10-fold greater selectivity for PLK1 compared to PLK2 or PLK3 was used to investigate combination effects with IPI-504. Treatment of H292 cells with an EC_50_ dose of IPI-504 for growth inhibition (175 nM) increased cell death from 24% when combined with vehicle to 45% when combined with an EC_50_ dose of BI2536 for growth inhibition (5 nM) (Fig. 2D and S1B Fig.). These data are consistent with the hypothesis that IPI-504 combines with different classes of antimitotics to promote cell death by independent mechanisms.

### IPI-504 and docetaxel combination treatment results in accumulation of mitotic cells

**Figure 3:**
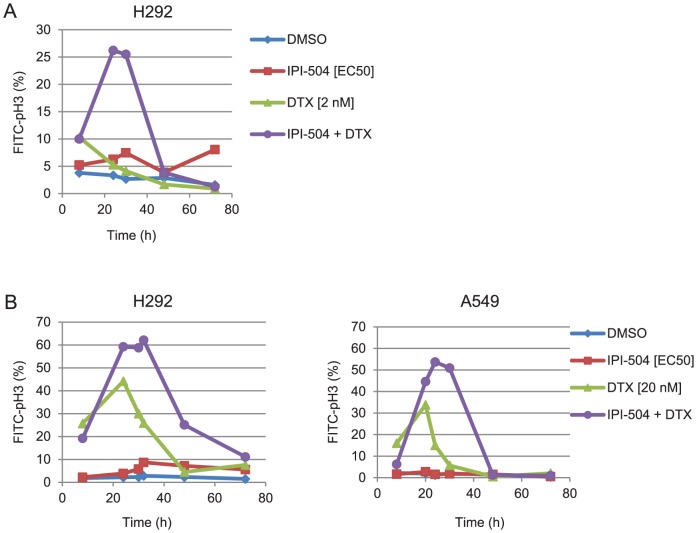
Mitotic delay upon treatment of cells with IPI-504 and docetaxel (DTX) combination. EC_50_ doses for IPI-504 were determined by 72 h Cell Titer Glo (S1A Fig.). H292 (A, B left panel) and A549 (B, right panel) cells were treated with DMSO (blue diamonds), IPI-504 (red squares), DTX (green triangles) or IPI-504/DTX combination (purple circles) and harvested at the indicated time points for presence of the mitotic marker, pH3. Representative data are shown (n = 2).

Given that the antimitotic effects of docetaxel are the molecular basis for its toxicity, we tested the prediction that IPI-504 enhances the antimitotic effects of docetaxel. The mitotic population, also known as mitotic index, was measured in H292 cells treated at various time points and dose combinations of IPI-504 and docetaxel. The combination of low dose (2 nM) docetaxel with IPI-504 resulted in a transient increase in mitotic index (10% at 8 h to 26% at 30 h) before returning to basal levels (4% at 48 h) (Fig. 3A). In contrast, the mitotic index did not rise above baseline throughout the duration of the experiment in cells treated with either IPI-504 or docetaxel as single agents (Fig. 3A). A similar transient increase in mitotic index followed by a decline was observed upon treating H292 cells with a high dose of docetaxel (20 nM) as a single agent (Fig. 3B, left panel). Addition of IPI-504 to high-dose docetaxel increased the amplitude and duration of the mitotic arrest (Fig. 3B, left panel). In contrast to H292 cells, an increase in mitotic index was not observed upon treatment of A549 cells with the low dose (2 nM) docetaxel and IPI-504 combination (data not shown), indicating that the response to this drug combination may be cell type specific. However, an increase in the amplitude and duration of mitotic arrest was observed in A549 cells treated with the high dose docetaxel (20 nM) and IPI-504 combination relative to docetaxel alone, comparable to that observed in H292 cells (Fig. 3B, right panel). Since the mitotic effects observed in combination with IPI-504 were consistent across multiple cell types, high-dose docetaxel was chosen for subsequent MOA studies.

### The mitotic checkpoint is partially responsible for the cell death effects of IPI-504 and docetaxel

**Figure 4:**
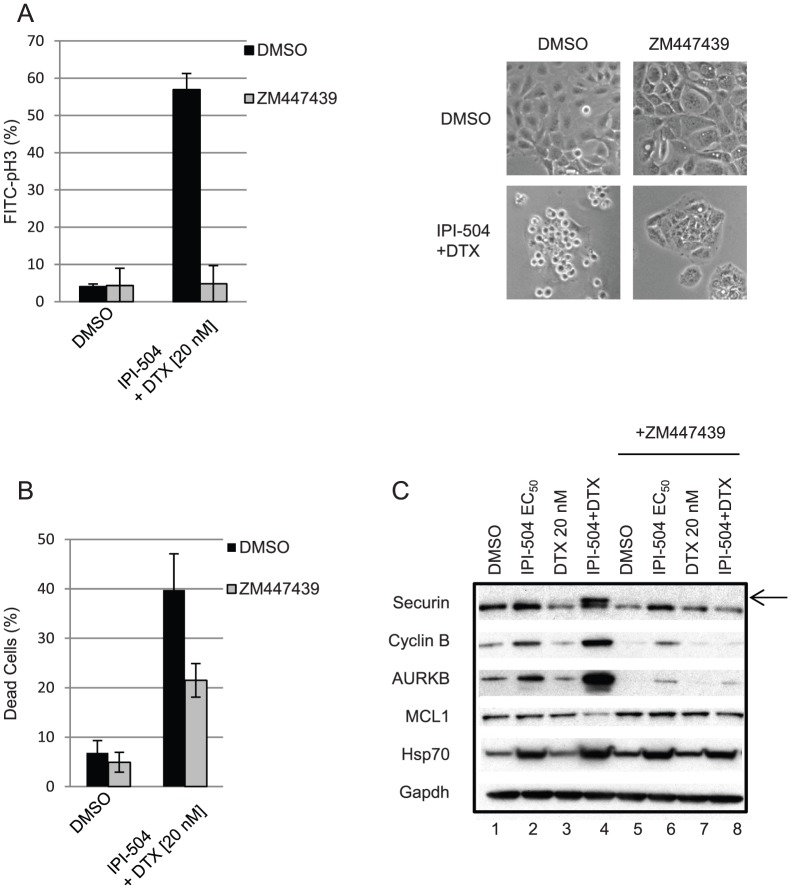
Mitotic checkpoint bypass with Aurora kinase inhibition partially rescues combination effects of IPI-504 and docetaxel (DTX). H292 cells were treated for 30 h (A, C) or 48 h (B) with indicated dose combinations of IPI-504 (175 nM), DTX (20 nM) and the Aurora kinase inhibitor ZM447439 (9 µM). Cells were harvested for (A) flow cytometry (pH 3) and phase contrast imaging, (B) cell death (7AAD), and (C) immunoblot analysis. An arrow denotes a slow migrating, phosphorylated form of Securin. Error bars for mitotic index and cell death represent standard deviation of replicates from two independent experiments.

The mitotic arrest preceding cell death in IPI-504 and docetaxel-treated cells suggested that activation of the mitotic checkpoint is an important determinant of the cell death response. To test this hypothesis, a selective Aurora A/Aurora B kinase inhibitor (ZM447439) was used to override the mitotic checkpoint in H292 cells treated with the IPI-504 and docetaxel combination. Treatment of H292 cells with ZM447439 resulted in abrogation of the IPI-504 and docetaxel-induced mitotic checkpoint arrest as determined by a marked decrease in the mitotic index (Fig. 4A, left panel). Phase contrast images provided visual confirmation of rounded up (mitotic) cells, prominently featured in cultures from IPI-504 and docetaxel treated cells as compared to the more flattened morphology of IPI-504, docetaxel, and ZM447439 treated cells (Fig. 4A, right panel). To evaluate the effect of mitotic checkpoint override, cell death was measured after treatment of H292 cells with IPI-504 and docetaxel in the presence or absence of ZM447439 (Fig. 4B). While the effects of Aurora kinase inhibition on cell death showed variable effects when combined with either IPI-504 or docetaxel as single agents (S2 Fig.), treatment with ZM447439 partially rescued the cell death synergy of IPI-504 and docetaxel, decreasing the percentage of dead cells from 40% to 22% (Fig. 4B). This is consistent with the hypothesis that the mitotic checkpoint is partially responsible for this synergistic effect.

Concomitant with mitotic arrest, IPI-504 and docetaxel dose combinations led to an accumulation of the mitotic proteins Securin, Cyclin B, and AURKB (Fig. 4C). The appearance of a slow migrating form of Securin, thought to represent the active, phosphorylated form [16] was observed specifically in cells treated with the IPI-504 and docetaxel combination (Fig. 4C, arrow). The slow migrating form was converted to the fast migrating form upon phosphatase treatment, confirming that the slow migrating form represents the phosphorylated form (S3 Fig.). Abrogation of the IPI-504 and docetaxel induced up-regulation of mitotic proteins Securin, Cyclin B, and AURKB was observed upon co-treatment with ZM447439 (Fig. 4C). In contrast, co-treatment with ZM447439 did not abrogate IPI-504 induced up-regulation of HSP70, a surrogate marker for HSP90 inhibition [17], indicating that IPI-504 is still active in these cells.

It has been reported that during prolonged mitotic arrest, phosphorylation of the anti-apoptotic protein MCL1 by Cyclin B-CDK1 initiates its APC/C-dependent destruction, leading to cell death during mitosis [18]. Interestingly, down-regulation of MCL1 protein was observed specifically in H292 cells treated with the IPI-504 and docetaxel combination, an effect that was abrogated upon co-treatment with the Aurora kinase inhibitor (Fig. 4C).

### Anaphase promoting complex (APC/C) components are specifically depleted from the HSP90 interactome in IPI-504 and docetaxel treated cells

**Figure 5:**
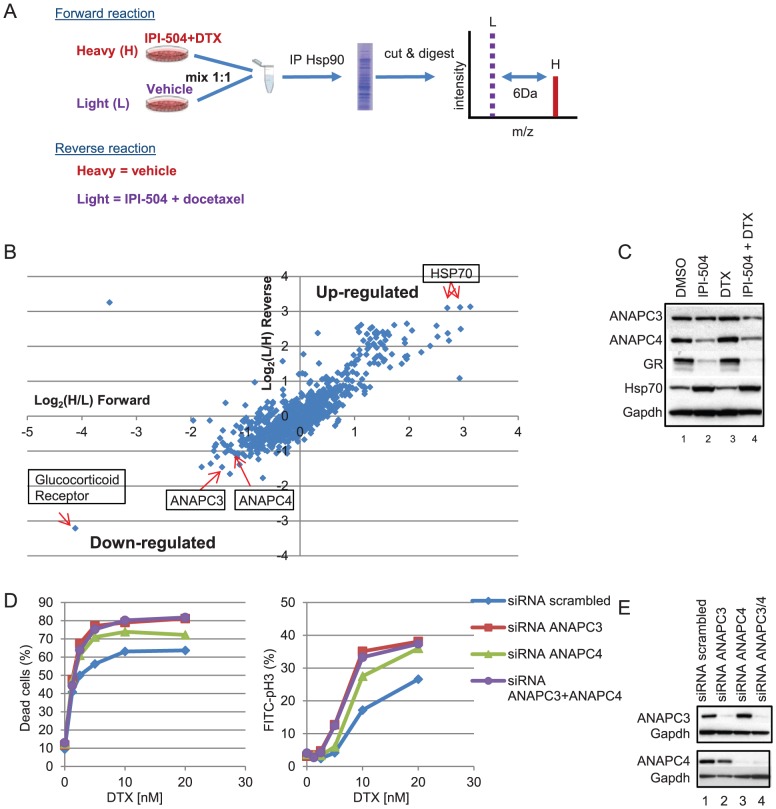
Identification of components of the anaphase promoting complex as specifically depleted from H292 cells treated with IPI-504 and docetaxel (DTX) combination. (A) Stable Isotope Labeling of Amino Acids in Culture (SILAC) schematic. Metabolically labeled H292 cells were treated for 24 h with the combination of 300 nM IPI-504 and 10 nM DTX or vehicle (DMSO) followed by identification of the HSP90 interactome by mass spectrometry. (B) Raw data from the SILAC study. Data points in the upper right and lower left quadrants represent proteins that are up-regulated and down-regulated, respectively in the HSP90 interactome upon treatment with the combination as compared to vehicle. The two arrows in the upper right quadrant represent independent peptide fragments with unique sequences targeting HSP70. (C) Immunoblot analysis verifies depletion of ANAPC3 and ANAPC4 upon 24 h treatment of H292 cells with 300 nM IPI-504 and 10 nM DTX combination. GR  =  Glucocorticoid Receptor. (D) Loss of APC/C components by RNAi sensitizes H292 cells to DTX mediated cell death (72 h, 7AAD) (left panel) and increases the mitotic index (30 h, pH 3) (right panel). H292 cells were transfected with nontargeting scrambled siRNA (blue diamonds) or siRNA's targeting ANAPC3 (red squares), ANAPC4 (green triangles), or ANAPC3/ANAPC4 combination (purple circles). (E) Immunoblot analysis showing knockdown efficiency. Representative data are shown (n = 2).

Upon inhibition of HSP90, misfolded client proteins dissociate from HSP90 and subsequently are targeted for proteasome-mediated degradation [19]. To identify potential client proteins that contribute to the synergy of IPI-504 and docetaxel, SILAC was performed and HSP90 interacting proteins comprising the “interactome” were identified under conditions of drug treatment by mass spectrometry. The HSP90 interactome for the forward experiment in which H292 cells labelled with heavy isotopes of lysine and arginine were treated with the combination of IPI-504 and docetaxel and H292 cells labelled with normal lysine and arginine were treated with vehicle alone was examined and compared to data obtained from the reverse experiment in which H292 cells labelled with the heavy isotopes were treated with vehicle alone and H292 cells labelled with the normal isotopes were treated with the combination of IPI-504 and docetaxel (Fig. 5A). As expected, Hsp70 was strongly up-regulated in the HSP90 interactome upon IPI-504 and docetaxel treatment (Fig. 5B) [17]. Likewise, Glucocortocoid Receptor (GR), a known HSP90 client protein, was strongly down-regulated from the HSP90 interactome upon combination treatment (Fig. 5B) [20]. Several potential novel HSP90 client proteins were identified that were down-regulated in response to the combination, a number of which play a role in the mitotic checkpoint response (S1 Table). These included two components of the APC/C, ANAPC3 and ANAPC4 (Fig. 5B). To confirm the SILAC data, protein abundance was determined by western blot analysis of lysates collected from H292 cells treated with IPI-504 and docetaxel in combination (Fig. 5C; S4 Fig.). Similar to GR, down-regulation of both ANAPC3 and ANAPC4 was observed upon treatment of H292 with the combination of IPI-504 and docetaxel compared to vehicle (Fig. 5C). The up-regulation of Hsp70 was confirmed upon treatment with IPI-504 alone or in combination with docetaxel (Fig. 5C).The contribution of APC/C to cell death synergy with the combination of IPI-504 and docetaxel was assessed by examining whether loss of APC/C components could replace IPI-504 in sensitizing cells to docetaxel. The APC/C components ANAPC3 and ANAPC4 were knocked down with siRNA individually or in combination, and the effects on cell proliferation were measured following docetaxel treatment. At concentrations of docetaxel greater than 5 nM, the plateau of cell death increased from 63% in the scrambled siRNA control, to 73% upon ANAPC4 knockdown, and 80% upon ANAPC3 knockdown alone or in combination with ANAPC4 knockdown (Fig. 5D, left panel). Loss of APC/C components enhanced the antimitotic effects of docetaxel, increasing the mitotic index from 17% with docetaxel (10 nM) alone to 33% when combined with ANAPC3/ANAPC4 loss (Fig. 5D, right panel). Western blot analysis confirmed efficient knockdown of APC/C components (Fig. 5E).

## Discussion

MTAs that disrupt microtubule dynamics are among the most effective antimitotic treatments for a broad range of cancers. Unfortunately, MTA-related toxicities and drug resistance remain major challenges. The identification of potential combination therapies could lead to increased efficacy, and allow for dose reductions that might reduce toxicity. Inhibition of the molecular chaperone, HSP90, is one candidate approach for combination therapy with antimitotics that has been evaluated in NSCLC clinical trials. We identified several NSCLC cell lines that showed in vitro and in vivo cell growth inhibition synergy in response to the combination of an HSP90 inhibitor, IPI-504, and the antimitotic MTA, docetaxel, when compared to these single agents alone. This enabled the exploration of the molecular basis of this drug synergy. Inhibition of PLK1, a mitotic regulator reported to require HSP90 chaperone activity, with BI2536 sensitized cells to IPI-504, suggesting that synergy with IPI-504 may not be restricted to the MTA class of antimitotics. This suggests that more efficacious combinations of HSP90 inhibitors and antimitotics may be identified, with improved side effect profiles compared to combinations with docetaxel.

HSP90 has been reported to regulate the metaphase to anaphase transition [21], therefore we hypothesized that inhibition of HSP90 would enhance the antimitotic effects of docetaxel. Consistent with this hypothesis, we observed a dramatic increase in the mitotic index upon treatment of NSCLC cells with the combination of IPI-504 and docetaxel at doses where neither drug had single agent activity. In vitro, docetaxel has different phenotypic consequences on the cell cycle depending on the dose administered. Low dose (1 to 4 nM range) docetaxel interferes with microtubule dynamics but is not sufficient to fully activate the checkpoint, resulting in aberrant mitotic exit, missegregation of chromosomes and aneuploidy [22], [23]. Higher doses (>10 nM) of docetaxel stabilize microtubules and arrest cells in G2/M, followed either by mitotic cell death or mitotic slippage into a tetraploid G1 [22], [23]. Treatment of H292 cells with IPI-504 produced antimitotic effects when combined with either low- or high-dose docetaxel, consistent with a common mechanism of action for the two dose combinations. It is noteworthy that the enhanced antimitotic effects of combining IPI-504 with doses of docetaxel sufficient to trigger the checkpoint were more consistent across cell types.

**Figure 6:**
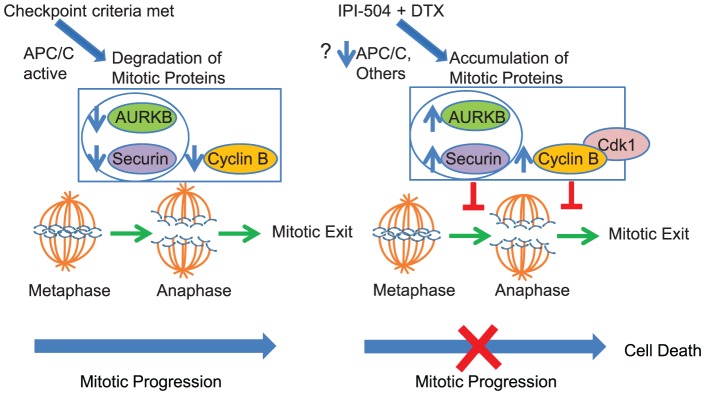
Model for IPI-504 and docetaxel (DTX) MOA. In the absence of drug treatment, the mitotic checkpoint criteria are met and APC/C dependent degradation of mitotic inhibitors Securin, AURKB, and Cyclin B allow cells to progress through mitosis (left panel). IPI-504 and DTX combination treatment results in accumulation of mitotic inhibitors and impaired mitotic progression (right panel). Elevated levels of AURKB and Securin block the metaphase to anaphase transition whereas elevated levels of Cyclin B block mitotic exit. The potential contributions of APC/C as an HSP90 client protein, or other HSP90 client proteins to the mitotic arrest observed upon combination treatment are indicated with a question mark.

These molecular MOA studies led us to propose the model illustrated in Fig. 6. This schematic depicts the molecular events preceding mitotic progression in untreated cells and cells treated with the IPI-504 and docetaxel combination. In untreated cells, the checkpoint criteria are met once chromosomes align along the metaphase plate with the proper spindle tension. This triggers a cascade of events involving disassembly of the mitotic checkpoint complex (MCC) and subsequent activation of APC/C, a multi-component E3 ubiquitin ligase responsible for the regulated degradation of mitotic inhibitor proteins Securin, Cyclin B, and AURKB [24]. APC/C binds to two substrate receptor proteins that aid in substrate recognition. Binding of APC/C to CDC20 is required for regulated turnover of Securin and Cyclin B, whereas binding of APC/C to CDH1 is responsible for regulated turnover of AURKB, a mitotic checkpoint regulator responsible for monitoring the attachment of microtubules to kinetochores [25]. Degradation of Securin results in the release of active Separase, a protease responsible for cleaving the SCC1 subunit of Cohesin which triggers sister chromatid separation [26]. Degradation of Cyclin B is required for mitotic exit [27]. By these means, APC/C dependent degradation of mitotic inhibitors allows cells to progress through mitosis. In contrast, accumulation of these proteins, experimentally observed in cells treated with IPI-504 and docetaxel, would be predicted to impede mitotic progression through AURKB-dependent activation of the checkpoint, Securin-mediated inhibition of sister chromatid separation, and cyclin B-mediated inhibition of mitotic exit.

Using an unbiased SILAC approach, we identified components of APC/C (ANAPC3 and ANAPC4) that were specifically depleted from the HSP90 interactome in cells treated with the IPI-504 and docetaxel combination, consistent with the accumulation of APC/C substrates observed in these cells. Whether down-regulation of APC/C components or other HSP90 client proteins are causal events in the accumulation of mitotic inhibitors in cells treated with the combination of IPI-504 and docetaxel has yet to be determined. We hypothesize that ANAPC3 and ANAPC4 represent novel HSP90 client proteins important for survival under conditions of mitotic stress such as docetaxel treatment. In support of this hypothesis, experimentally-induced loss of APC/C components (ANAPC3, ANAPC4) enhanced the antimitotic effects of docetaxel and sensitized cells to docetaxel-induced cell death; however, the relatively weak effects indicate that additional factors would be required to explain the in vitro synergy.

Numerous reports have demonstrated that having an intact mitotic checkpoint response is a key determinant of cellular sensitivity to docetaxel [28]. Our data indicate that the mitotic checkpoint is a key determinant of cellular sensitivity to IPI-504 and docetaxel in combination as well. Specifically, abrogation of the checkpoint with an inhibitor of Aurora kinase A and Aurora kinase B (ZM447439) partially rescued the cell death effects of the IPI-504 and docetaxel combination. Further experiments using more selective Aurora kinase inhibitors would be required to address whether the ability of ZM447439 to abrogate the checkpoint is attributable to its inhibitory activity towards a particular kinase.

During prolonged mitosis, Cyclin B-CDK1 dependent phosphorylation of the anti-apoptotic protein MCL1 creates a recognition site for APC/C-dependent degradation [18]. We hypothesize that the down-regulation of MCL1, observed in cells treated with IPI-504 and docetaxel, may be a contributing factor to the cell death response. In support of this hypothesis, abrogation of the mitotic checkpoint with the Aurora kinase inhibitor ZM447439 partially rescued the cell death effects as well as the MCL1 down-regulation observed upon combination treatment. Further studies to determine the contribution of MCL1 or other BCL2 family members to the cell death response will help elucidate the molecular details of IPI-504 and docetaxel drug synergy and may help explain the differences in sensitivity to drug combination among cell types with differential expression of various apoptotic family members.

One challenge facing HSP90 inhibitor therapy is the compensatory upregulation of HSP70. Inhibitor binding to HSP90 causes dissociation of active Heat Shock Factor 1 and subsequent transcriptional upregulation of HSP70 which can attenuate the cytotoxic effects of HSP90 inhibitors [17]. In a recent report, X-ray irradiation has been shown to enhance the effects of the HSP90 inhibitor, NVP-BEF800 on glioblastoma through a mechanism involving attenuation of HSP70 upregulation [29]. As predicted, we observed upregulation of HSP70 bound to HSP90 upon treatment of cells with IPI-504. Treatment of H292 cells with the combination of docetaxel and IPI-504 did not abrogate the compensatory HSP70 upregulation, indicating that the growth inhibitory effects of this particular combination occur through an alternative mechanism, despite the elevated levels of HSP70.

In summary, our data suggest that HSP90 inhibition in combination with agents that prolong mitosis or prevent mitotic exit have synergistic effects in in vivo and in vitro models of NSCLC. Antimitotics exert their cytotoxic effects primarily during mitosis. Therefore, lengthening the duration of mitosis would increase the percentage of cells poised to optimally respond to drug treatment. Mitosis is the shortest phase of the cell cycle, typically lasting 15 min to 1 hour and can only be extended up to 1 to 2 days after which Cyclin B levels begin to decline and cells exit mitosis despite having not met the checkpoint criteria [30]. Certain cell types are able to escape the cytotoxic effects of antimitotics by prematurely slipping out of mitosis [31], [32]. Preventing mitotic slippage by knocking down CDC20 leads to the accumulation of Cyclin B, checkpoint independent mitotic arrest, and cell death [33]. This work has led to the emerging hypothesis that blocking mitotic exit may be a better therapeutic strategy than interfering with microtubule dynamics, particularly in slippage-prone cells. We have shown that inhibition of HSP90 is a promising strategy in combination with docetaxel, through a mechanism involving the lengthening of mitosis. Our model predicts that the accumulation of Cyclin B observed upon treatment of cells with IPI-504 and docetaxel prevents mitotic exit. As such, inhibition of HSP90 could be a good therapeutic strategy in combination with antimitotics to specifically target slippage-prone cells. Future studies comparing different classes of antimitotics in combination with HSP90 inhibitors will help determine whether there is a correlation between the duration of mitotic arrest and cell death synergy. Given the heterogeneity of cellular responses to antimitotics, a deeper understanding of the consequences of combining these agents with HSP90 inhibitors will require more sophisticated methods for cell fate determination such as live cell imaging. Further mechanistic insight into the molecular mechanism of action will help guide the path forward for the development of rational combination therapies for the treatment of patients with NSCLC and other malignancies.

## Supporting Information

S1 Fig **Growth inhibition curves.** (A) H292, H1993, and A549 cells were treated with IPI-504 for 72 h; cell growth inhibition was measured by Cell Titer Glo. EC_20_, EC_50_, and EC_80_ values were calculated using KaleidaGraph V3.52 software. (B) H292 cells were treated for 72 h with the PLK1 inhibitor, BI2536; cell growth inhibition was measured by Cell Titer Glo. The EC_50_ value was calculated using GraphPad Prism V6.01. Error bars represent standard deviation (n = 2).
(EPS)

S2 Fig **Cell death responses of IPI-504 or docetaxel (DTX) in combination with Aurora kinase inhibitor ZM447439.** H292 cells were treated for 48 h with IPI-504 (175 nM) or DTX (20 nM) in combination with AURORA kinase inhibitor ZM447439 (9 µM). Cell death was measured by Cell Titer Glo. Results represent the average of two independent experiments and error bars represent standard deviation.
(EPS)

S3 Fig **Presence of a slow mobility, phosphorylated form of Securin in the mitotic cell population.** A549 cells were treated with the indicated doses of IPI-504 and docetaxel (DTX) for 51 h. A549 cells treated with the combination of IPI-504 and DTX were separated into mitotic and nonmitotic (adherent) populations by mitotic shake-off. Mitotic cells were lysed in RIPA buffer and incubated in the presence or absence of alkaline phosphatase at 37°C for 30 min. Arrow indicates slow mobility, phosphorylated form of Securin that is lost upon treatment with alkaline phosphatase.
(EPS)

S4 Fig **Down-regulation of anaphase promoting complex components, ANAPC3 and ANAPC4 upon treatment with multiple dose combinations of IPI-504 and docetaxel (DTX).** H292 cells were harvested 24 h post drug treatment with the indicated dose combinations of IPI-504 and docetaxel followed by immunoblot analysis.
(EPS)

S1 Table **Raw data from SILAC study.** Values corresponding to each protein are listed as Log2 ratios of (H/L) for the forward experiment in which heavy-labeled cells were treated with IPI-504 (300 nM) and docetaxel (10 nM) combination and light-labeled cells were treated with vehicle and (L/H) for the reverse experiment in which heavy labeled cells were treated with vehicle and light labeled cells were treated with IPI-504 (300 nM) and docetaxel (10 nM) combination.
(XLSX)
